# Electrophysiology in neuropathic pain: a bibliometric analysis and literature review

**DOI:** 10.3389/fnins.2025.1616973

**Published:** 2025-06-03

**Authors:** Yidan Cui, Chen Lv, Wenjian Yan, Lidan Zhang, Ning Sun, Xin Zhang, Zhen Zhong

**Affiliations:** College of Acupuncture and Massage, Changchun University of Chinese Medicine, Changchun, Jilin, China

**Keywords:** neuropathic pain, electrophysiology, bibliometrics, CiteSpace, VOSviewer

## Abstract

**Objective:**

Neuropathic pain (NP), a prevalent chronic condition with increasing global incidence, mainly relies on electrophysiology (EP) to decode its mechanisms. However, existing research lacks systematic integration, failing to track hotspots and frontiers effectively. In this study, we used bibliometric analysis and systematic review to clarify technological breakthrough directions and facilitate the development of electrophysiological phenotype-based precision diagnostics.

**Methods:**

Literature was retrieved from the Web of Science Core Collection (WoSCC). A total of 2,234 reviews and articles were obtained from 2005 to 2024. Statistics and visualization analysis were performed using Bibliometrix (R), VOSviewer, CiteSpace, and Microsoft Excel 2024.

**Results:**

Publications and citations in this field are rising. The USA leads in publications (613, 27.44%) and academic impact (H-index = 96). China’s academic impact remains behind when compared to other countries. North American and Western European institutions form robust collaboration networks, whereas Asian institutions exhibit weaker regional partnerships. Authors with high production, such as Khanna, Rajesh and Waxman, Stephen G, and highly cited authors such as Woolf, CJ play a key leading role in the development of the field. Journals like Pain and Journal of Neuroscience are the cardinal dissemination mediums. Keyword analysis reveals research hotspot expands from the basic structure of “dorsal root ganglion” and” sensory neuron” to pain-related dynamic changes and disease prevalence characteristics. “Plasticity” and “connectivity” signaled a shift in research toward network mechanisms and precise interventions. “Woolf CJ, 2011” and “Dib-Hajj SD, 2010” have high citation and co-citation frequencies. The emergence of new directions such as “dynamic pain connectome” and “computational modeling research” reflects the trend of multidisciplinary integration.

**Conclusion:**

For the first time, we have constructed a complete lineage from basic research to clinical translation in this field, confirming the key role of EP technology in analyzing the mechanism of nociceptive sensitization, neuroplasticity, and neural network connectivity reconfiguration, which provides a basis for developing precise diagnostic and therapeutic solutions based on electrophysiological phenotypes. Future research should focus on technology standardization, cross-institutional data sharing, clinical translation, and connectomics-oriented individualized analgesic strategies to promote NP diagnosis and treatment toward precision, dynamics, and systematization.

## 1 Introduction

The International Association for the Study of Pain (IASP) defines neuropathic pain (NP) as “pain caused by a lesion or disease of the somatosensory nervous system” (Haanpää et al., 2011). A cross-sectional survey that included 148,828 individuals showed the prevalence of NP was 9.2% in the general population (Baskozos et al., 2023), which seriously affects the health and quality of life of patients. Recent meta-analyses have shown that only 30–40% of patients with NP respond well to drug therapy (Finnerup et al., 2018; Moisset et al., 2020). In addition, it is challenging to develop drugs against new targets for NP (Rice et al., 2014, 2021; Attal and Bouhassira, 2019; Bouhassira and Attal, 2018). Considering the limitations of drugs, neurostimulation techniques such as spinal cord electrical stimulation (SCS) and transcranial magnetic stimulation (TMS) have been widely explored (Knotkova et al., 2021; Garcia-Larrea and Quesada, 2022). However, the existing studies are biased in the assessment of efficacy due to sample heterogeneity and insufficient standardization of parameters (Attal et al., 2023). Therefore, individualized treatment has become the key to breaking through the bottleneck (Baron et al., 2012; Baron et al., 2023; Edwards et al., 2023). Electrophysiology (EP), as a core technology in neuroscience, can resolve the mechanisms of neural signal generation, transmission, and regulation at the cellular and molecular levels through bioelectrical signals (Peterson et al., 2023; Shen et al., 2023; Song et al., 2024; Jiang et al., 2024). In recent years, some studies published in Nature have utilized advanced EP techniques such as membrane clamp and single-cell recording to deeply explore the ion channel function and neuronal excitability alteration associated with neuralgia, laying the foundation for precise interventions based on electrophysiological phenotypes (Draper-Joyce et al., 2021; Chen et al., 2024; Kim et al., 2024). However, there is a lack of systematic integration of current research results, key technological breakthroughs have failed to effectively promote clinical applications, and interdisciplinary integration has not yet formed a scale. This study combed the research lineage of EP in NP through bibliometric visualization and systematic review, which can grasp the research hotspots and frontiers, aiming to clarify the direction of technological breakthroughs. It provides a basis for developing electrophysiological phenotype-based precision diagnosis and treatment programs.

## 2 Materials and methods

### 2.1 Data sources and search strategies

To ensure the quality of the literature and to follow the proper reference format, the Science Citation Index-Expanded in the Web of Science Core Collection (WoSCC) was selected as the data source for this study. WoSCC contains more than 12,000 high-quality scientific journals, which is reliable and is regarded as the best database for bibliometric studies (Durieux and Gevenois, 2010; Santos et al., 2020). This study focuses on the use of EP in NP research over the past 20 years, and is based on a full-year analysis cycle; therefore, data from 2025 were not included. The search was conducted on February 11, 2025, by two researchers (YiDan Cui and Chen Lv) independently.

In terms of search terms, keyword #1 for data collection was a different expression of “neuropathic pain,” covering acronyms, standardized terms, and patient descriptors. Keyword #2 was the electrophysiology section, including core concepts of underlying mechanisms, experimental methods, and signal detection techniques. Both sets of search terms utilize the wildcard character “*” to expand derivatives, lexemes, and singular plurals. The specific search strategy is as follows:

#1: TS = (“NP” OR “neuropathic pain” OR “neuralgia*” OR “neurodynia*” OR “neurogenic pain” OR “neuralgic pain” OR “nerve pain*”).

#2: TS = (“electrophysiolog*” OR “bioelectric*” OR “membrane potential” OR “action potential” OR “ion channel*” OR “patch clamp” OR “voltage clamp” OR “current clamp” OR “intracellular recording” OR “extracellular recording” OR “single-unit recording” OR “local field potential” OR “EEG” OR “electroencephalogra*” OR “ECG” OR “EKG” OR “electrocardiogra*” OR “EMG” OR “electromyogra*” OR “evoked potential” OR “ERP” OR “event-related potential”).

Final: #1 AND #2.

The final publication year is limited to 2005–2024, the language is English, the publication type is limited to “review” and “article,” and the field of study is limited to “Neurosciences Neurology.” The final search yielded a total of 2,234 publications.

### 2.2 Data extraction and collection

We downloaded and exported all search results via “Export - Plain text file - Full Record and Cited References.” It contains all the necessary information such as title, year of publication, number of citations, author, journal, funding agency, field of study, author keywords, and references. For the statistics, we determined the country/region of origin of the article based on the nationality of its first author, and defined “collaboration” as papers involving authors from at least two different countries/institutions. The impact factor (IF) and quartile rankings of journals by subject category were obtained from the 2023 Journal Citation Reports (JCR). The H-index is defined as the number of papers (h) that have received at least h citations, which is often used to measure the cumulative impact of a country’s/institution’s output. Total link strength (TLS) indicates the sum of the strength of the co-citation relationship between an author and all other cited authors. The mediator centrality (BC) of a node is the ratio of the number of shortest paths through that node to the number of shortest paths of all node pairs. A high BC value indicates that the node plays a key role in mediating information transfer, resource flow, etc. in the network.

### 2.3 Bibliometric analysis and statistical analysis

Bibliometrix: the Bibliometrix R software package allows for the analysis of developments in the field of research, quantitative assessment of research results. We used it to analyze collaboration among countries and annual publication trends in producing countries (Aria and Cuccurullo, 2017).

VOSviewer: using VOSviewer 1.6.20, we flexibly set the thresholds according to the characteristics and needs of the analysis project to generate visual charts for collaborative network analysis, and realize collaborative analysis of institutions/authors/journals, co-citation analysis of authors, and co-occurrence analysis of keywords. In addition, clusters are generated by grouping keywords based on their relevance, and temporal overlay analysis is completed by assigning different colors to keywords according to the average year in which they appear (van Eck and Waltman, 2010, 2014; Donthu et al., 2021).

CiteSpace: we used CiteSpace (6.4.R1, 64-bit Advanced), and scientifically optimized the parameters based on the research objectives and data characteristics. Its algorithmic burst detection captures sharp increases in the popularity of references or keywords over a specified period, and the burst “strength” is an indicator that measures the degree of the surge in its citation frequency. Knowledge maps for co-authorship analysis of institutions, cross-referencing of disciplines in journals, the bursts of keywords and references, and clusters analysis of references were successfully drawn. Modularity value (Q) > 0.3 and Silhouette value (S) > 0.7 imply a significant and convincing cluster structure (Chen, 2006).

Microsoft Excel 2024: we used it for descriptive statistical analysis and graphing, fitting curves to the number of publications and citations per year and selecting the best-fitting model based on the highest correlation coefficient (R^2^).

## 3 Results

### 3.1 Analysis of annual publication and citation trends

**Figure 1:**
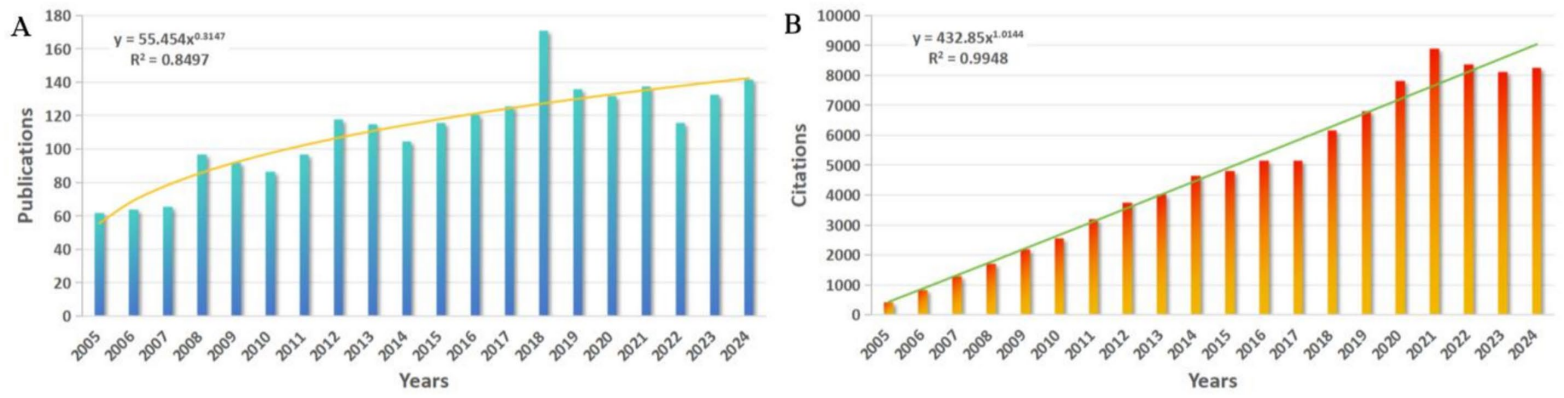
**(A)** Number of annual publications and fitted curves. **(B)** Annual citation frequency and fitted curves.

From 2005 to 2024, a total of 2,234 publications in the WoSCC database, including 1,973 Articles (88.32%) and 261 Reviews (11.68%). The total number of citations for papers is 88,584 (83,019 without self-citation), with an average of 39.65 citations per paper and an H-index of 123. The number of publications per year (Figure 1A) increases from 62 in 2005 to 142 in 2024, with a power function fit curve showing an increasing trend over the last 20 years (R^2^ = 0.8497). Correspondingly, the number of citations per year (Figure 1B), which is also fitted using a power function, shows a more significant increasing trend (R^2^ = 0.9948), steadily increasing from 444 in 2005 to 8,265 in 2024. Figure 1A shows a significant rise in publications in 2018, representing a 35.71% increase compared to 2017. The development slowed down after 2019, the epidemic is undoubtedly one of the important influencing factors.

### 3.2 Analysis of national publication volume and collaboration

**Figure 2:**
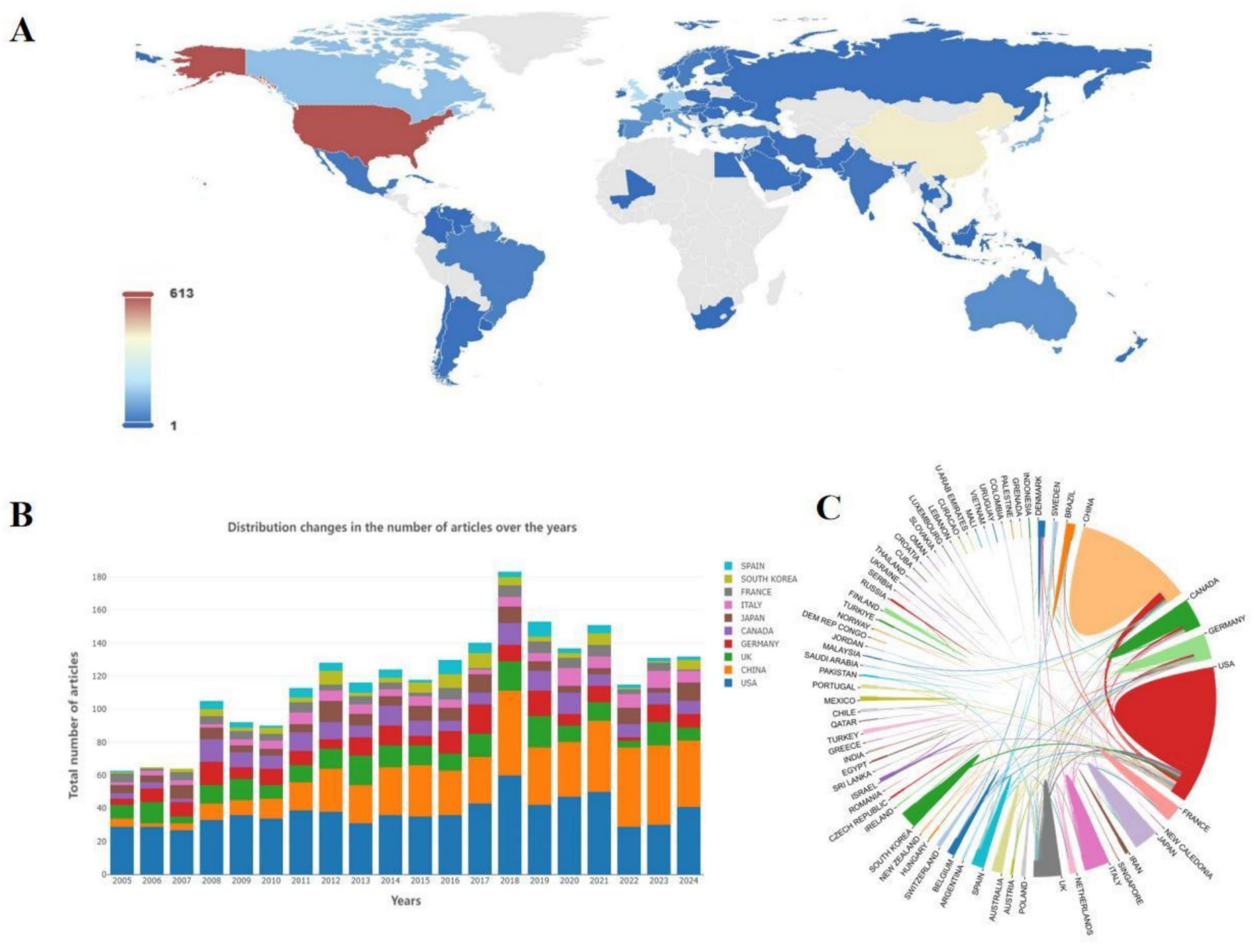
**(A)** Schematic diagram of the world distribution of the number of national publications. **(B)** Schematic diagram of the distribution and changes in the number of publications in different countries by year. **(C)** Chord diagram of the cooperation relationship among countries.

Table 1 and Figure 2A show 71 countries with contributing articles, with the USA (613, 27.44%) first, China (428, 19.16%) second, and the UK (167, 7.47%) third. In terms of academic influence measured by the H-index (Lü et al., 2016), the USA (H-index = 96) also ranked first. However, China (H-index = 49) is significantly lower than the UK (H-index = 60), indicating that China’s scientific research strength and influence in this field still need to be improved.

**Table 1:** Top 10 countries with the most publications.

Rank	Country	Publications	% of 2,234	H-index
1	USA	613	27.44	96
2	China	428	19.16	49
3	UK	167	7.47	60
4	Germany	156	6.98	45
5	Canada	139	6.22	47
6	Japan	113	5.06	39
7	Italy	90	4.03	39
8	France	73	3.28	33
9	Korea	64	2.86	27
10	Spain	61	2.73	25
	Others	330	14.77	

Figure 2B shows that the USA has produced steadily since the early stages. In contrast, China has grown rapidly and even outpaced the USA recently though later entering, which is expected to explore the field in depth and produce more high-quality results. The USA has been extremely active in country cooperation (Figure 2C), participating in numerous cooperation programs as an initiating country. The USA collaborates most frequently with China (91 times) and maintains high-frequency partnerships with the UK, Canada, and Korea. Overall, cross-continental cooperation is more common, which promotes knowledge exchange and technology sharing exhibitions between different regions.

### 3.3 Contribution of institutions

The number of publications, average number of citations per article, and H-index of an institution are important indicators of its research strength and influence. A total of 2,148 organizations were included in this study. The statistics are consolidated based on institutional affiliation and different expressions for the same institution. For example, “University College London” was merged into “University of London,” “Harvard University Medical Affiliates” was merged into “Harvard University,” and “Veterans Health Administration (VHA)” was merged into “US Department of Veterans Affairs.” The top 10 institutions in terms of the number of publications (Table 2) are from different countries such as the UK, the US, France, and Canada. Among them, University of London (92, 4.118%) is at the top of the list and also has the highest H-index = 42 among the 10 institutions, indicating that a considerable number of papers have been frequently cited within a certain period of time. US Department of Veterans Affairs has an average of 245.27 citations per article, and Harvard University has an average of 234.06 citations per article, which far exceeded those of the other institutions, indicating that the quality and innovativeness of their research results are widely recognized.

**Table 2:** Top 10 institutions with the most publications.

Rank	Institutions	Total publications	% of 2,234	Citations per paper	H-index
1	University of London	92	4.118	62.29	42
2	University of Texas System	83	3.715	59.96	38
3	University of California System	65	2.91	51.37	29
4	Yale University	62	2.775	83.95	33
5	Institut National de la Sante et de la Recherche Medicale Inserm	55	2.462	51.31	26
6	Harvard University	52	2.328	234.06	33
7	University of Toronto	52	2.328	76.31	31
8	US Department of Veterans Affairs	49	2.193	245.27	32
9	Centre National de la Recherche Scientifique CNRS	42	1.88	43.17	23
10	VA Connecticut Healthcare System	40	1.791	83.48	25

**Figure 3:**
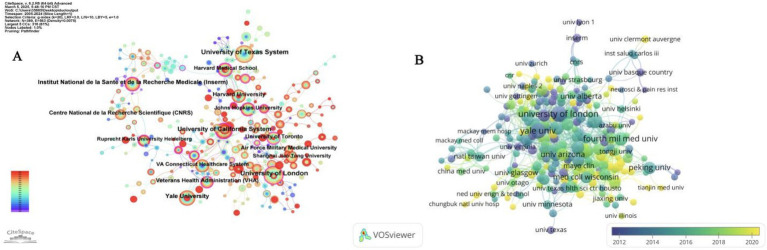
**(A)** Analysis of cooperation between institutions. **(B)** Collaborative relationships between institutions overlaid on a timeline visualization map.

The analysis of institutional collaboration was conducted by CiteSpace (Figure 3A), and the node size in the figure is proportional to the number of articles. It can be seen that the collaboration among institutions is mainly concentrated in countries in North America and Western Europe. Like the University of California System, the University of London and other nodes have more connections, forming a complex cooperation network. While some institutions in Asian countries, such as Shanghai Jiao Tong University, have contributed to the number of articles, the cooperation network among institutions within the region has not yet been formed, and academic exchanges in this field have yet to be strengthened. In addition, among all these institutions, the University of Toronto has the highest centrality (BC value = 0.25), reflecting its importance in information transfer and resource sharing in the network.

Figure 3B is a network diagram of institutional collaborations generated by VOSviewer, in which the nodes are marked with different colors according to their average year of appearance. According to the indication, “University of London” and “Yale University,” which are shown as purple nodes, are relatively early entrants into the research field; while institutions with yellow nodes such as “Tianjin Medical University” and “University Clermont Auvergne” are relatively new entrants. This shows that most of the Chinese research institutions entered the field late and there is still room for improvement in the current level of cooperation. However, “Fourth Military Medical University,” as the institution with the highest citation frequency in China, shows certain unique advantages and influence.

### 3.4 Authors and co-cited authors

**Figure 4:**
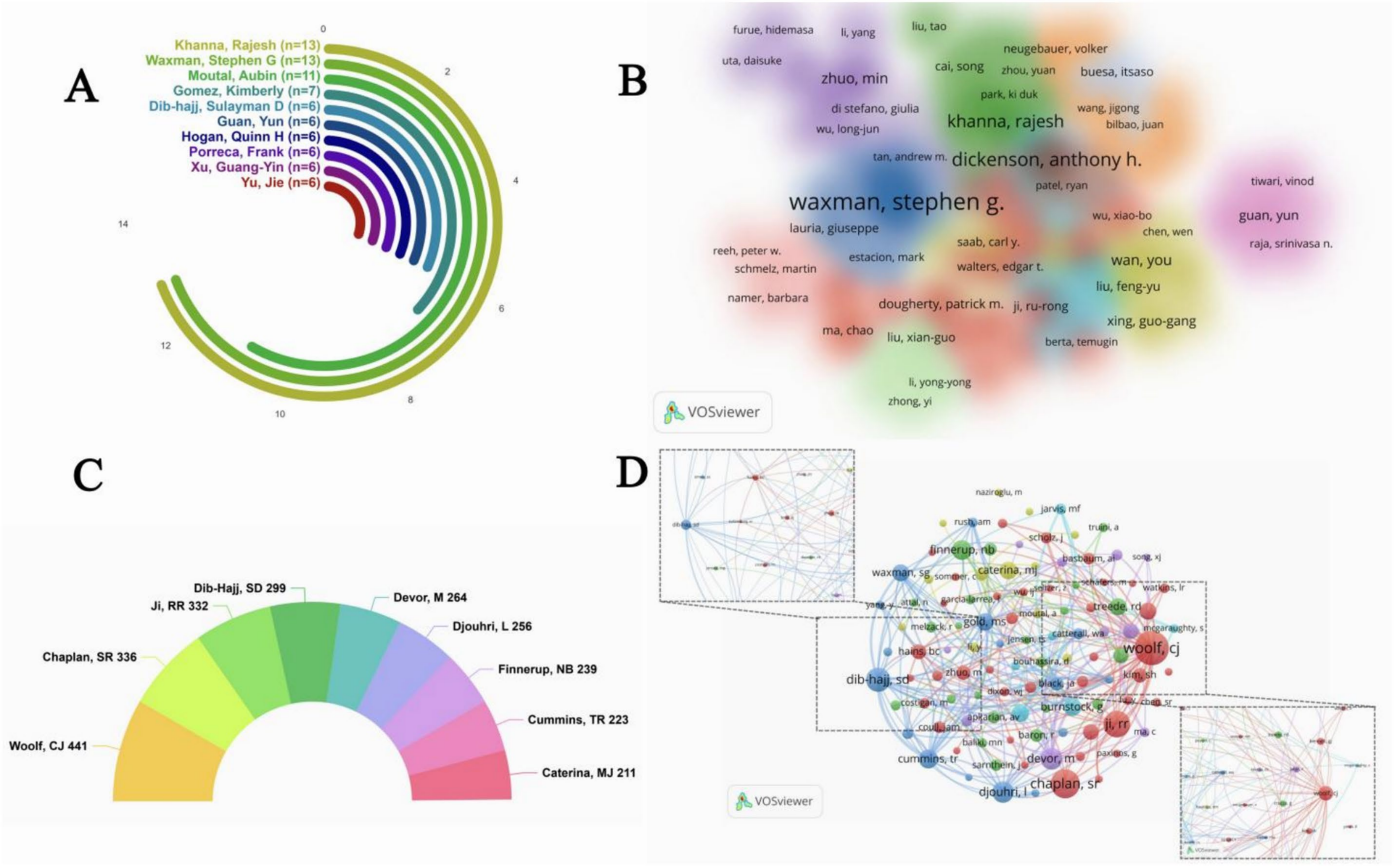
**(A)** Top 10 authors with the most publications. **(B)** Cluster analysis of the co-author relationships among the authors. **(C)** Top 10 authors with the most co-citations. **(D)** Visualization map of author co-citation analysis.

A total of 9,586 authors were involved in publishing articles in NP and EP studies. Among the top 10 most productive authors (Figure 4A), Khanna, Rajesh and Waxman, Stephen G both published 13 papers and are at the top of the list, followed by Moutal, Aubin with 11 publications.

The cluster analysis of author collaboration, drawn by VOSviewer (Figure 4B), reveals that different colored areas represent multiple research clusters. Within each cluster, authors are closely connected, with core authors acting as radial points to conduct collaborative research. In the figure, Waxman, Stephen G. and Dickenson, Anthony H. are in the relative core position. Waxman, Stephen G. (Persson et al., 2016; Zakrzewska et al., 2017; Alsaloum and Waxman, 2022; Ovsepian and Waxman, 2023; Baron et al., 2023; Fu et al., 2024) is a professor at Harvard Medical School in the United States, with in-depth research in the field of neuroscience, especially in neural axon EP and molecular mechanisms of neurological diseases. Dickenson, Anthony H. (Baron et al., 2023; Bannister et al., 2020; Yosten et al., 2020) specializes in pain research and has made important contributions in areas such as the pathogenesis of NP. The region of color interweaving is relatively small in the whole figure, implying that there is not yet a widespread trend of cross-cluster cooperation.

A co-citation relationship means that two authors or articles appear simultaneously in the reference list of a third document. Author co-citation analysis based on this is often used to reveal key authors within a specific field (Navarro-Ballester et al., 2023; Trujillo and Long, 2018). Among the top 10 co-cited authors (Figure 4C), Woolf, CJ tops the list with 441 co-citations, followed by Chaplan, SR with 336 co-citations.

In addition, a higher TLS value means that the author has more academic connections and influence in the field. From the author co-citation visualization network (Figure 4D), Woolf, CJ (red node, TSL = 17,604) is at the core of the network, followed by Dib-Hajj, SD (blue node, TSL = 12,471) and Ji, RR (TSL = 11,263).

### 3.5 Journals and co-cited journals

The present analysis covers 258 journals, and Table 3 lists the top 10 journals in terms of the number of articles published. There are 4 journals in both the UK and the USA, reflecting the well-established academic evaluation systems and sufficient publication channels in the field in both countries. IF is a key indicator of the academic impact of journals. These 10 journals have an IF of 2.1–5.9, and half of the journals in the Q1 division have high authority and influence in this field. Taking all the indicators together, Pain has the most outstanding performance, with advantages in the number of published articles (219), IF (5.9), and division (Q1), making it a highly influential journal in the field.

**Table 3:** Top 10 journals with the most output.

Rank	Journals	Output	% of 2,234	Country	IF (2023)	JCR quartile
1	Pain	219	9.80	USA	5.9	Q1
2	Molecular Pain	129	5.77	UK	2.8	Q2
3	Journal of Neuroscience	93	4.16	USA	4.4	Q1
4	Neuroscience	81	3.63	UK	2.9	Q2
5	Neuropharmacology	74	3.31	UK	4.6	Q1
6	Brain Research	57	2.55	Netherlands	2.7	Q3
7	Journal of Neurophysiology	55	2.46	USA	2.1	Q3
8	Experimental Neurology	47	2.10	USA	4.6	Q1
9	Neuroscience Letters	47	2.10	Netherlands	2.5	Q3
10	European Journal Of Pain	45	2.01	UK	3.5	Q1

**Figure 5:**
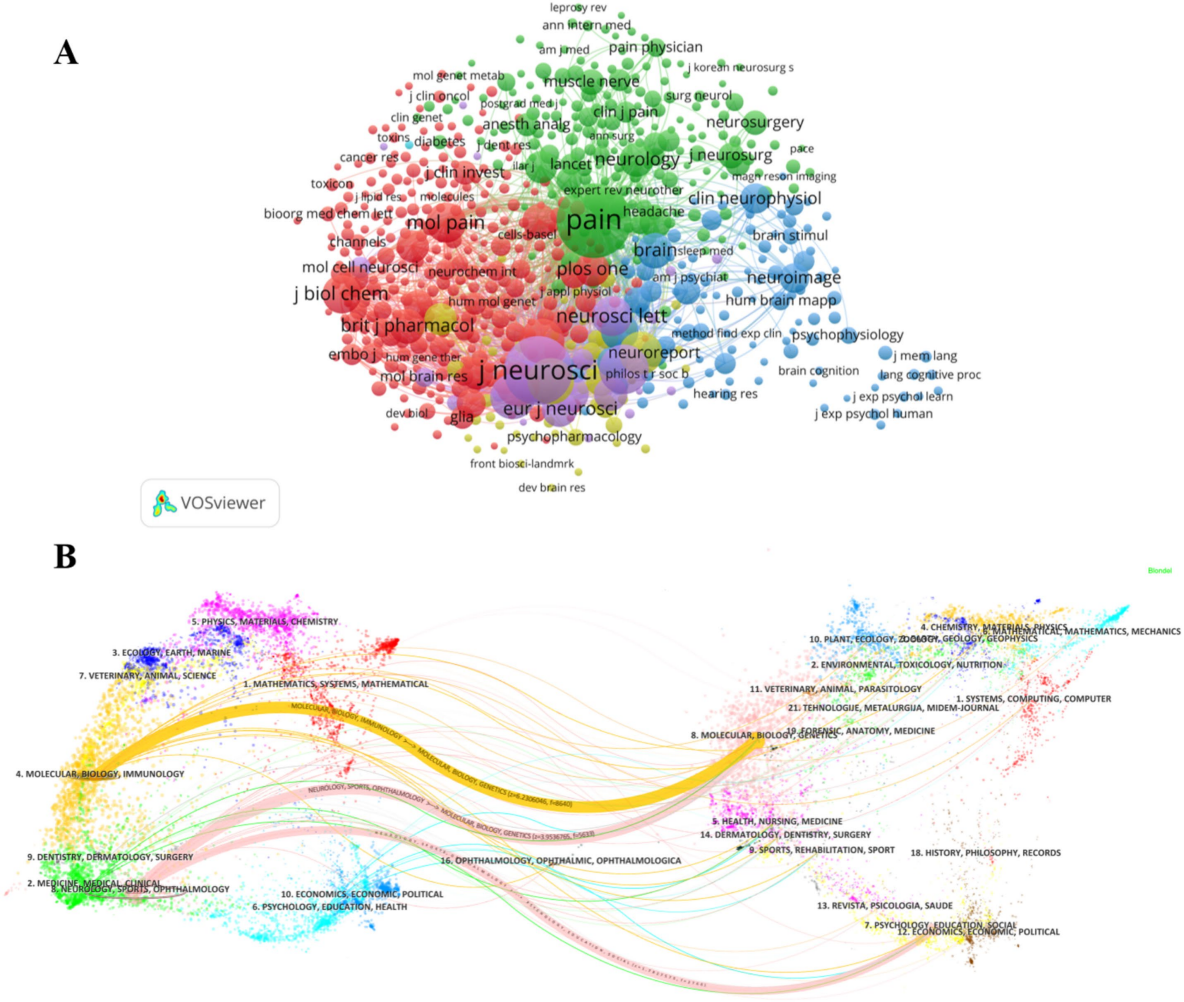
**(A)** Co-citation analysis of the journals. **(B)** The dual-map overlay of journals related to Electrophysiology and neuropathic pain.

In this study, journal co-citation analysis (Figure 5A) was performed using VOSviewer with a minimum citation threshold of 20 citations, generating a network graph with 667 nodes, 6 clusters, and 135,880 links. The top 5 co-cited journals are Pain (10,404), Journal of Neuroscience (8,338), Journal of Neurophysiology (3,509), Neuroscience (2,927), Journal of Physiology-London (2,746), which are important vehicles for knowledge dissemination and academic exchange in this field.

Pain focuses on the pathophysiological mechanisms of pain and clinical diagnosis and treatment strategies; Neuroscience and Journal of Neuroscience focus on the frontiers and hotspots in the field of neuroscience; Journal of Neurophysiology and Journal of Physiology-London are mainly devoted to neuromodulation, cellular electrophysiology and other physiological mechanisms. In addition, we can see the disciplinary crossover and citation relationships from the double-mapped overlay layer (Figure 5B). For example, the yellow thick path shows that literature published in Molecular/Biology/Immunology journals often cites literature published in Molecular/Biology/Genetics journals. The pink thick path shows that literature published in Neurology/Sports/Ophthalmology journals tends to cite literature published in Molecular/Biology/Genetics and Psychology/Education/Social journals.

It highlights the fundamental position of Molecular/Biology/Genetics disciplines in multidisciplinary research and also shows that there are potential research links between different fields.

### 3.6 Keywords co-occurrence analysis

**Figure 6:**
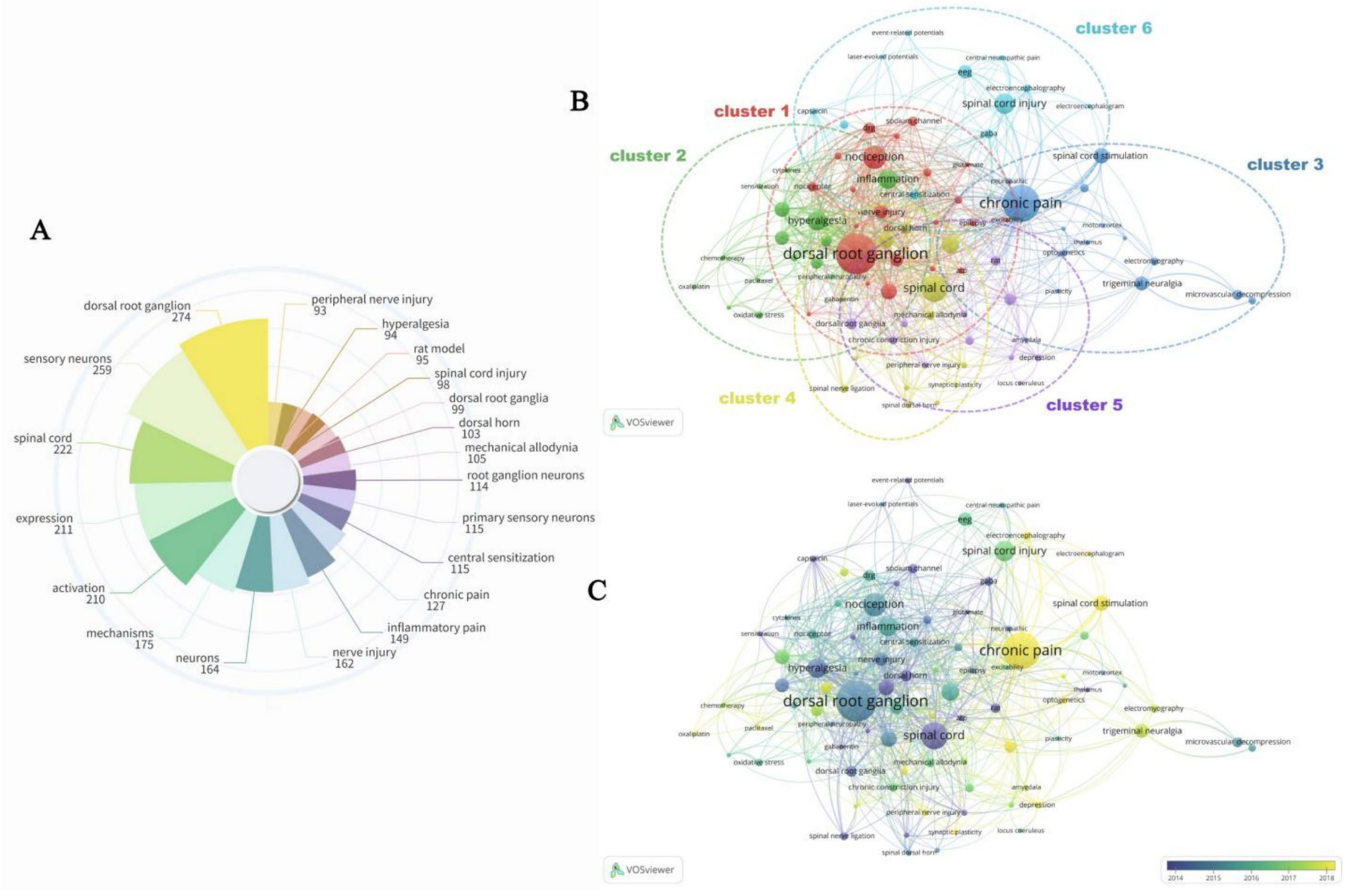
**(A)** Top 25 keywords with the largest occurrence times. **(B)** Network visualization map of keywords co-occurrence analysis. **(C)** Overlay timeline visual map for keyword co-occurrence analysis.

The present study collected all the author keywords in each article totaling 9, 208, representing the major themes and core content. Figure 6A shows the top 20 keywords in order of frequency of occurrence. “Dorsal root ganglion (DRG)” appeared most frequently (*n* = 274), serving as a key anatomical node and core target for nerve conduction. It was followed by “sensory neurons” (*n* = 259) and “spinal cord” (*n* = 222). The “rat model” (*n* = 95) is one of the only animal experimental tools used to simulate human neurological and pain-related conditions and plays a key role in basic research and drug testing. The “mechanical allodynia” (*n* = 105) and “inflammatory pain” (*n* = 149) provide directions for the analysis of pain mechanisms and the development of analgesic programs from the perspective of pain triggers.

Keyword co-occurrence analysis was performed using VOSviewer which demonstrates the frequency of keyword usage relationship strength and co-occurrence frequency by the size of the dots the distance between the dots and the thickness of the lines. All keywords were categorized into six clusters (Figure 6B): Cluster 1 (red node basic research on neurophysiology and pain): Contains “action potential,” “analgesia,” “dorsal root ganglion,” etc.; cluster 2 (green node disease and inflammation related research): “Chemotherapy,” “cytokines,” “hyperalgesia,” etc.; cluster 3 (dark blue nodes neuromodulation and clinical applications): “Deep brain stimulation,” “electromyography,” “neuromodulation,” etc.; cluster 4 (yellow nodes sensory neurons and pain perception research): Like “allodynia,” “dorsal horn,” “*in vivo* electrophysiology,” etc.; cluster 5 (purple nodes neuroanatomy and disease correlation research): “Amygdala,” “anterior cingulate cortex,” “chronic pain,” etc.; cluster 6 (light blue nodes neurophysiological tests and technical studies): “EEG,” “electroencephalogram,” “spinal cord injury,” etc. through the temporal overlap analysis network (Figure 6C) it can be seen that the purple or blue nodes represent the keywords that appeared earlier and the yellow nodes are the current research focus which helps to grasp the development of the research in the field and hotspots

### 3.7 Keywords burst analysis

**Figure 7:**
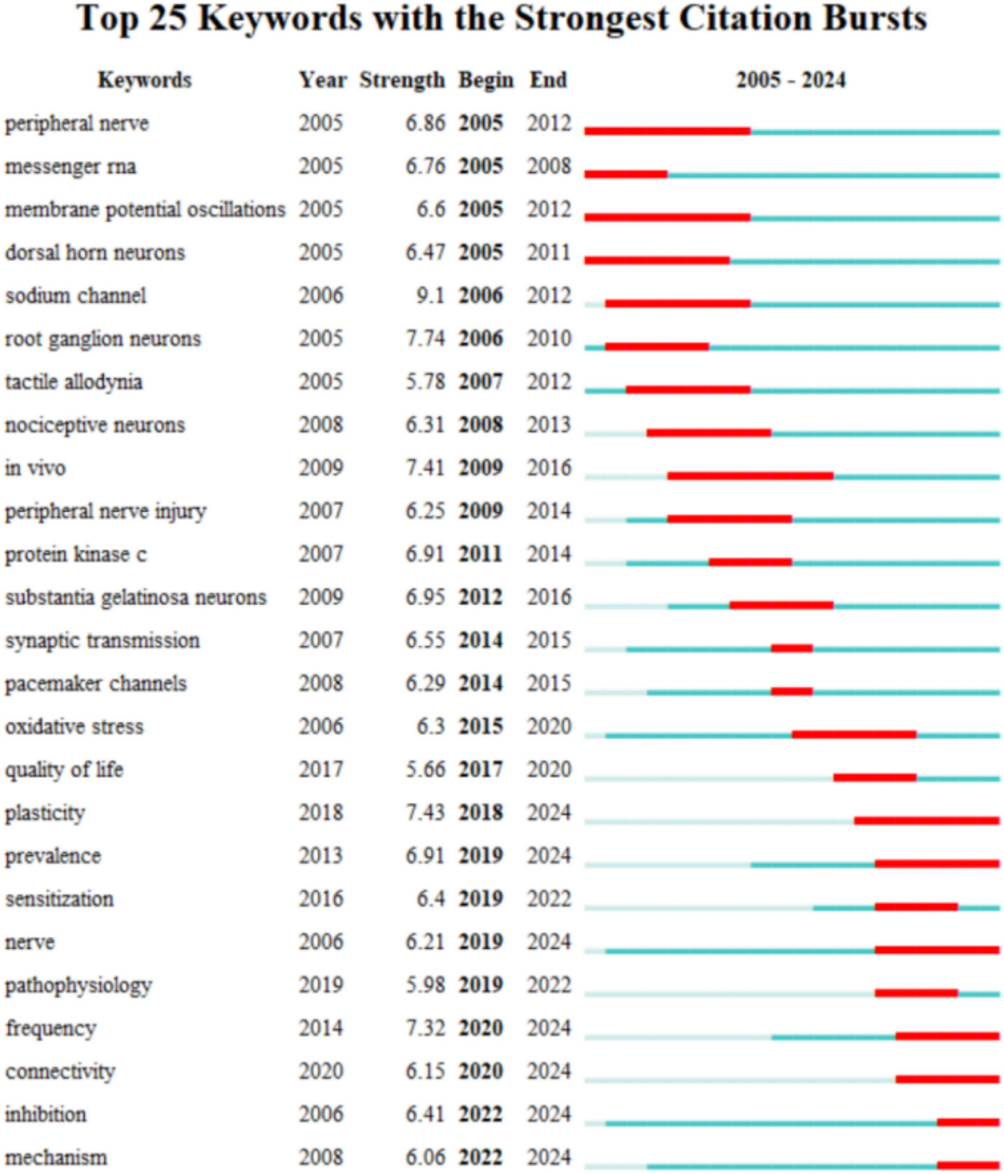
Top 25 keywords with the strongest citation bursts.

Using Citespace to detect emerging keywords, we screened the top 25 keywords with the strongest citation bursts (Figure 7). We can see that “peripheral nerve” (2005–2012), “membrane potential oscillations” (2005–2012), and “in vivo” (2009–2016) have received the most sustained attention (8 years each) over time. In addition, the keywords that appear after 2018 and continue to 2024 are “plasticity” (2018–2024, strength 7.43), “prevalence” (2019–2024, strength 6.91), “nerve” (2019–2024, strength 6.21), “frequency” (2020–2024, strength 7.32), “connectivity” (2020–2024, strong 6.15), “inhibition” (2022–2024, strong 6.41), and “mechanism” (2022–2024, strong 6.06), indicating that these keywords have become more attractive in recent years and represent popular research topics in recent years and even soon.

### 3.8 References and co-cited references

Citation analysis is one of the key methods in bibliometrics, where the number of citations measures the impact of a publication and the level of scholarship in the field. Table 4 shows the top 20 highly cited articles in EP in NP, with a total citation frequency of ranging from 308 to 3,017. For example, “Woolf CJ, 2011“(Woolf, 2011) (3,017 citations, average of 201.13 citations per year) continues to receive attention as it provides critical support for diagnosis and treatment. Early literature such as “Chessell, IP, 2005″ (Chessell et al., 2005) (655 citations, average of 31.19 citations per year) laid the groundwork for the development of the field through genetic studies, and subsequent studies continue to be cited to delve deeper into the mechanisms involved. Articles published after 2010, such as “Woolf CJ, 2011” (Woolf, 2011) and “Bih, Clementino Ibeas, 2015″ (Ibeas Bih et al., 2015), have higher total citations and average annual citation frequency. It reflects that new results in this field are emerging, gaining high attention and recognition, and the research heat continues to rise. Among these high-frequency cited documents, JOURNAL OF NEUROSCIENCE issued four articles, PAIN issued three articles, and NEURON and BRAIN issued two articles each, which also reflects the important power of these journals to promote the development of the discipline.

**Table 4:** Top 20 most cited references.

Rank	Cited references	Total citations	Average per year	First author	Source title	Publication year
1	Central sensitization: Implications for the diagnosis and treatment of pain	3,017	201.13	Woolf CJ	Pain	2011
2	Central Sensitization: A Generator of Pain Hypersensitivity by Central Neural Plasticity	2,533	149	Latremoliere, Alban	JOURNAL OF PAIN	2009
3	TRPA1 contributes to cold, mechanical, and chemical Nociception but is not essential for hair-cell transduction	1,017	50.85	Kwan, KY	Neuron	2006
4	Cytokine mechanisms of central sensitization: Distinct and overlapping role of interleukin-1β, interleukin-6, and tumor necrosis factor-β in regulating synaptic and neuronal activity in the superficial spinal cord	939	52.17	Kawasaki, Yasuhiko	Journal of Neuroscience	2008
5	Disruption of the P2X_7_ purinoceptor gene abolishes chronic inflammatory and neuropathic pain	655	31.19	Chessell, IP	Pain	2005
6	Attenuated cold sensitivity in TRPM8 null mice	581	30.58	Colburn, Raymond W	Neuron	2007
7	The diagnostic criteria for small fibre neuropathy: from symptoms to neuropathology	557	30.94	Devigili, Grazia	Brain	2008
8	Activated microglia contribute to the maintenance of chronic pain after spinal cord injury	532	26.6	Hains, BC	Journal of Neuroscience	2006
9	Sodium Channels in Normal and Pathological Pain	490	30.63	Dib-Hajj SD	Annual Review of Neuroscience	2010
10	JNK-Induced MCP-1 Production in Spinal Cord Astrocytes Contributes to Central Sensitization and Neuropathic Pain	476	28	Gao, Yong-Jing	Journal of Neuroscience	2009
11	The Na_V_1.7 sodium channel: from molecule to man	441	33.92	Dib-Hajj SD	Nature Reviews Neuroscience	2013
12	Molecular Targets of Cannabidiol in Neurological Disorders	404	36.73	Bih, Clementino Ibeas	Neurotherapeutics	2015
13	The roles of sodium channels in nociception: Implications for mechanisms of pain	374	19.68	Cummins, Theodore R	Pain	2007
14	HC-030031, a TRPA1 selective antagonist, attenuates inflammatory- and neuropathy-induced mechanical hypersensitivity	363	20.17	Eid, Samer R	Molecular Pain	2008
15	Increased EEG power and slowed dominant frequency in patients with neurogenic pain	332	16.6	Sarnthein, J	Brain	2006
16	Purinergic signalling: From normal behavior to pathological brain function	329	21.93	Burnstock, Geoffrey	Progress in Neurobiology	2011
17	Chemotherapy-induced peripheral neuropathy: What do we know about mechanisms?	321	29.18	Carozzi, V. A	Neuroscience Letters	2015
18	Responsible, Safe, and Effective Prescription of Opioids for Chronic Non-Cancer Pain: American Society of Interventional Pain Physicians (ASIPP) Guidelines	311	34.56	Manchikanti, Laxmaiah	Pain Physician	2017
19	Presynaptic and postsynaptic amplifications of neuropathic pain in the anterior cingulate cortex	308	17.11	Xu, Hui	Journal of Neuroscience	2008
20	Motor cortex rTMS restores defective intracortical inhibition in chronic neuropathic pain	308	15.4	Lefaucheur, J. P	Neurology	2006

Table 5 summarizes the top 10 co-cited references and all authors published only 1 article. Among them, “Neuropathic pain” by Colloca et al. (2017) has the highest co-citation frequency (*n* = 41), and the journal in which it was published also has the highest IF (76.9). The publication years ranged from 2005 to 2019, with 2 articles appearing in 2011 and 1 article per year in the remaining years. Of the source journals, Physiol Rev published 2 articles, and the remaining journals all published only 1 article. There are two overlaps between the T0P10 highly cited literature (Table 4) and the T0P10 co-cited literature (Table 5), which are “Woolf CJ, 2011“(Woolf, 2011) (TOP1 cited, TOP7 co-cited) and “Dib-Hajj SD, 2010″ (Dib-Hajj et al., 2010) (TOP9 cited, TOP4 co-cited). It shows that these two documents are the core knowledge in the field, as well as fitting the research hotspots and occupying a key position in the knowledge network.

**Table 5:** Top 10 most co-cited references.

Rank	Co-cited references	Total co-citations	First author	Source title	IF (2023)	Publication year
1	Neuropathic pain	41	Colloca L	Nat Rev Dis Primers	76.9	2017
2	The Role of Voltage-Gated Sodium Channels in Pain Signaling	33	Bennett DL	Physiol Rev	29.9	2019
3	HCN2 Ion Channels Play a Central Role in Inflammatory and Neuropathic Pain	29	Emery EC	Science	44.7	2011
4	Sodium Channels in Normal and Pathological Pain	28	Dib-Hajj SD	Annu Rev Neurosci	12.1	2010
5	BDNF from microglia causes the shift in neuronal anion gradient underlying neuropathic pain	24	Coull JAM	Nature	50.5	2005
6	Pharmacotherapy for neuropathic pain in adults: a systematic review and meta-analysis	24	Finnerup NB	Lancet Neurol	46.5	2015
7	Central sensitization: Implications for the diagnosis and treatment of pain	24	Woolf CJ	Pain	5.9	2011
8	Mechanisms of neuropathic pain	24	Campbell JN	Neuron	14.7	2006
9	Regulating excitability of peripheral afferents: emerging ion channel targets	23	Waxman SG	Nat Neurosci	21.2	2014
10	Models and Mechanisms of Hyperalgesia and Allodynia	22	Sandkühler J	Physiol Rev	29.9	2009

**Figure 8:**
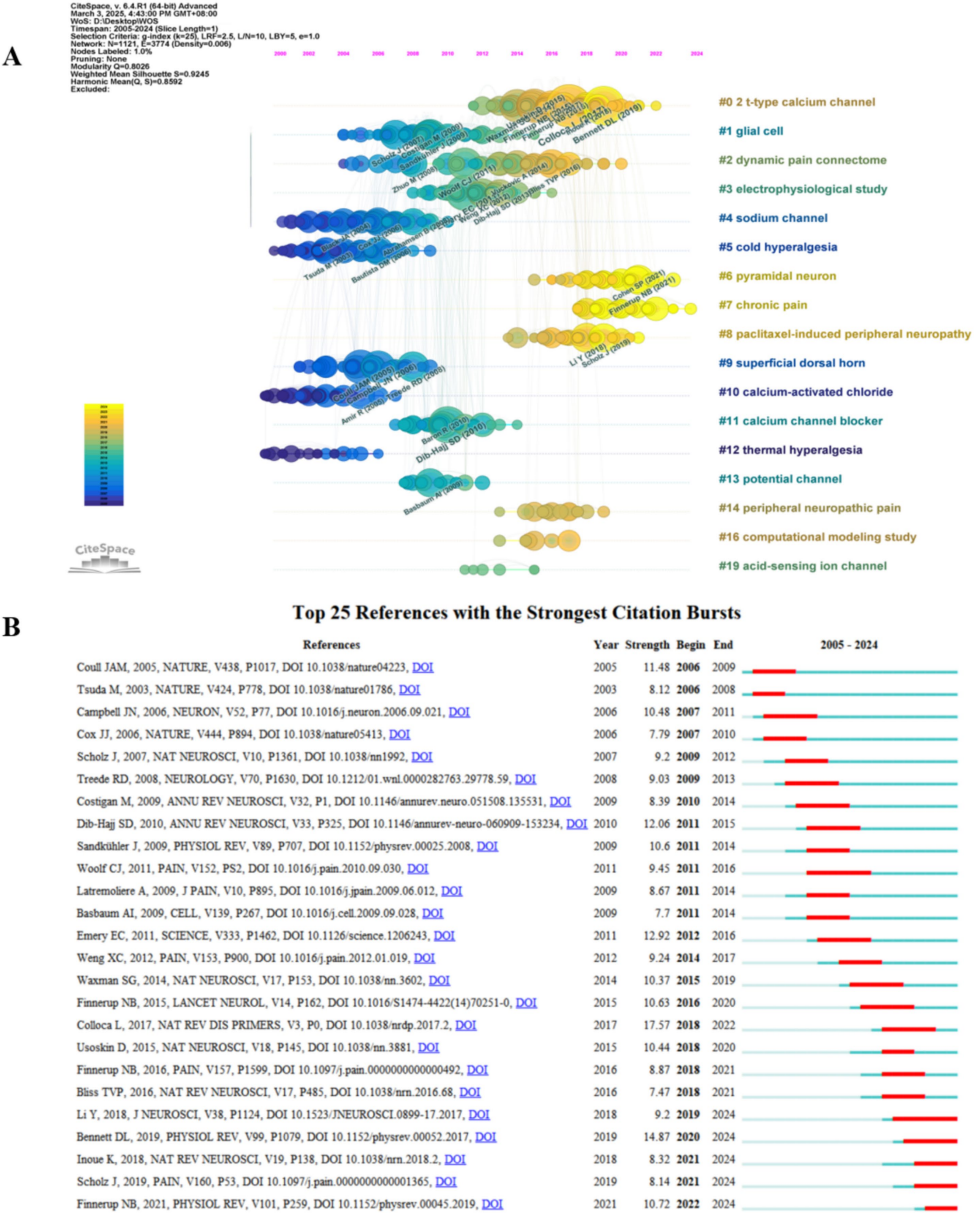
**(A)** Cluster timeline plot of the co-cited references. **(B)** Top 25 references with the strongest citation bursts.

The results of the co-cited reference network constructed by CiteSpace showed 19 clusters with Q = 0.8026 and S = 0.9245. Except for #1 and #3, the rest of the clusters with S > 0.91, possess good homogeneity. The evolutionary characteristics of each cluster can be seen from the timeline view of the clusters (Figure 8A). Early research hotspots such as “sodium channel” (#4) and “cold hyperalgesia” (#5) have many and large nodes in the early stages but change in heat in the later stages, implying that they will serve as a foundation for the development of the field that plays an underlying supportive role with emerging thematic convergence. Comparatively, “pyramidal neuron” (#6), “chronic pain” (#7), etc., still have more and larger nodes in the later part of the timeline, suggesting that their roles in relevant physiopathological processes remain the research focus in recent years. The emergence of clustering categories such as “dynamic pain connectome” (#2) signals the expansion of research to the level of neural network connectivity, which can help to reveal more comprehensively the mechanisms of pain onset and regulation. The emergence of research methodology categories such as “computational modeling study” (#16) signals a trend toward multidisciplinary cross-fertilization, where research aided by computer simulation and modeling will become more prevalent. The clustering of “paclitaxel-induced peripheral neuropathy” (#8) and “peripheral neuropathic pain” (#14) reflects the fact that research is moving away from broad domains and deepening into the direction of associating specific conditions with pain mechanisms, facilitating the development of targeted therapeutic programs.

Evaluating references with high citation explosion rates using CiteSpace is an important tool for mining research frontiers and hotspots. Among the Top 25 references with the strongest citation bursts (Figure 8B), “Bennett DL, 2019” (Bennett et al., 2019) (2019–2024, strength 14.87) and “Finnerup NB, 2021,” (Finnerup et al., 2021) (2022–2024, strength 10.72) are the references with the strongest bursts in recent years.

## 4 Discussion

Based on the bibliometric analysis, the research object of EP in the field of NP expands from a single target to the whole brain network, the technical means develops from the traditional ex vivo detection tools to the multimodal fusion *in vivo* monitoring, and the research direction shifts from the mechanism analysis to the clinical translation in the period of 2005–2024. The reason for the sharp rise in the number of publications in 2018 may be that 2016 through 2018 was a period of critical iteration in neurophysiological technology, such as the maturation of Adeno-Associated Virus (AAV) vector-targeted delivery technology (Pillay et al., 2016; Tabebordbar et al., 2016; Smalley, 2017; George et al., 2017), the application of Neuropixels probes (Jun et al., 2017; Steinmetz et al., 2018; Vélez-Fort et al., 2018), and the development of closed-loop SCS technology (Angeli et al., 2018; Wagner et al., 2018), which significantly improved the signal acquisition accuracy and clinical intervention efficacy. In addition, after the declaration of the opioid crisis in the United States (DeWeerdt, 2019; Brody, 2019; Eisenstein, 2019; Volkow and Koroshetz, 2019), the support of related policies has also injected a strong impetus for research. It is found that the United States and China, as the top two countries in terms of the number of articles published, have formed a differentiated academic influence in this field: the United States leads the field by its high H-index, while China needs to continue to improve. The network of institutional cooperation shows a pattern of coexistence between the North America-Western Europe core circle and the emerging power in Asia. The research direction also reflects the trend of multidisciplinary integration. It is possible that in research areas such as motor neuroscience and visual neuroscience, not only the basic theories of genetics are needed, but also the knowledge of psychology and sociology should be combined to explore the effects of exercise on the nervous system and the social factors of visual impairment. Through keyword analysis, we capture the dynamic evolution of research hotspots such as “dorsal root ganglion,” “plasticity,” “connectivity,” “frequency,” etc. Combined with the results of bibliometric visualization, we now develop a systematic discussion from the following perspectives.

### 4.1 EP is the core technology to resolve the mechanism of DRG nociceptive sensitization

“Dorsal root ganglion” was used as a high-frequency keyword throughout the study, confirming its central position as a nociceptive signaling hub. Among the high-frequency cited literature, the central sensitization theory proposed by Woolf (2011) lays a theoretical framework for DRG research, while Dib-Hajj SD’s (2010) study on the function of sodium channels provides a direct target for the analysis of molecular mechanisms. Electrophysiological recording, capable of capturing dynamic changes in neuronal membrane potential and ion channels, serves as a core tool for dissecting DRG pain sensitization mechanisms.

In a spinal nerve ligation model, the whole-cell current clamp technique showed decreased basal intensity, decreased action potential burst thresholds, and a significantly higher proportion of spontaneous discharges in medium- and small-diameter neurons of the DRG on the injured side, suggesting that increased neuronal excitability may be a direct cause of nociceptive sensitization (Xia et al., 2021). The membrane clamp technique further revealed that the absolute resting membrane potential of DRG neurons in rats with chronic pain was reduced, and the frequency of action potential firing and the amplitude of TRPV1 channel currents were elevated, suggesting that abnormal ion channel function plays an important role in pain signaling (Friston et al., 2023).

At the level of molecular mechanisms, electrophysiologic evidence of aberrant sodium channel expression provides a direct target for pain treatment. In a paclitaxel-induced model of peripheral neuropathic pain, mRNA and protein expression of the DRG neuron Nav1.7 was significantly upregulated, and inhibition of Nav1.7 attenuated pain symptoms by decreasing neuronal spontaneous firing (Xu et al., 2022). In addition, the expression of NALCN sodium leakage channel was increased in the chronic constriction injury (CCI) model, and its enhanced function could promote neuronal sensitization by modulating the background sodium leakage conductance, providing a new direction for targeted therapy (Zhang et al., 2021a).

The EP study also revealed a novel mechanism for the abnormal discharge of DRG neurons. *In vivo* imaging revealed synchronized aggregated discharges in adjacent DRG neurons after nerve injury, a discharge pattern mediated by sympathetically released norepinephrine via α2 adrenergic receptors, which is directly related to spontaneous pain behavior (Inyang et al., 2025). In addition, TRPM3 cation channels are highly expressed in DRG nociceptive neurons, whose activation triggers neurogenic inflammation and is involved in thermal nociceptive sensitization, while TRPM3 blockers have shown analgesic potential in animal models (Rodrigues et al., 2022).

In terms of clinical translation, DRG electrical stimulation technology has achieved significant efficacy in intractable pain such as complex regional pain syndrome and diabetic peripheral neuropathy by precisely modulating neuronal discharge. For example, dual-electrode implantation of SCS can simultaneously relieve post-stroke thalamic pain and limb paralysis, and its mechanism may be related to the inhibition of DRG abnormal discharge and remodeling of signaling in the dorsal horn of the spinal cord (Lee et al., 2024). These studies not only deepen the understanding of the pathological mechanisms of NP but also lay the foundation for the development of novel therapies targeting ion channels and neuromodulation.

### 4.2 EP is a key tool for studying the dynamic regulatory mechanisms of neuroplasticity

The keyword “plasticity” exploded with a strength of 7.43 after 2018, indicating that research in this field has entered the stage of precision regulation. In the co-cited literature, Colloca L’s (2017) discourse on pain plasticity forms theoretical support with Waxman SG’s (2014) ion channel research. EP technology provides a core experimental basis for elucidating the neuroplasticity mechanism of NP by tracking neuronal firing patterns, synaptic transmission efficacy, and network oscillation properties in real-time. Its collocation with optogenetics, molecular biology, and other technologies is driving the innovative development of non-invasive neuromodulation strategies.

In a mouse model, repetitive transcranial direct current stimulation (tDCS) of the prefrontal cortex reverses NP by neural remodeling: single stimulation elicits widespread cortical excitation, whereas repetitive stimulation induces large-scale silencing of cortical activity. This difference is closely related to altered activation patterns of GABAergic interneurons and excitatory neurons (Gan et al., 2021). After nerve injury, *β*-wave oscillations in the anterior cingulate cortex (ACC) are significantly enhanced, while the event-related desynchronization (ERD) response to tactile stimuli is more intense in the theta/alpha/beta frequency bands, and this oscillatory abnormality may be involved in the maintenance of nociceptive hypersensitivity (Mirmoosavi et al., 2024). In a model of resinoid toxin-induced neuropathy, α2δ-1 up-regulation of DRG led to a significant increase in the frequency of microexcitatory postsynaptic currents (mEPSCs) and the amplitude of evoked EPSCs in spinal cord dorsal horn neurons by augmenting NMDA receptor-mediated glutamatergic input. Blockade of NMDA receptors or inhibition of α2δ-1 reverses this effect (Zhang et al., 2021b).

Enhanced projections from the anterior cingulate cortex (ACC) to the dorsomedial striatum (DMS) are accompanied by abnormal synaptic plasticity in dopamine D1R neurons in a chronic pain model. Optogenetic and electrophysiologic recordings show that inhibition of this pathway alleviates pain-related insomnia (Li Y. D. et al., 2024). Multifocal tDCS-targeted motor networks reversed capsaicin-induced inhibition of N2-P2 cortical responses in persistent pain, implying the plastic modulatory potential of motor cortex-related networks in nociceptive processing (Gregoret et al., 2023).

### 4.3 Explanation of the cross-scale mechanism of neural connectome remodeling by EP

The keyword “connectivity” has a burst strength of 6.15 after 2020, indicating that the research focus has shifted to network mechanisms. In the co-citation analysis, the “dynamic pain connectome” clustering reveals the functional integration pattern of pain-related brain regions, which is highly compatible with the direction of connectomics research advocated in recent years. EP technology can realize the dynamic monitoring and functional verification of neural connectivity at the cross-scale from the single cell to the whole brain network. Through real-time recording of neuronal clusters’ firing patterns, oscillatory coupling characteristics, and cross-brain information transfer, it reveals the reconstruction pattern of pain-related neural circuits and lays a technical foundation for the development of precise analgesic strategies based on connectomics.

Pyramidal neurons in the CA1 region of the ventral hippocampus (vCA1) exhibited functional heterogeneity in nociceptive coding, and their enhanced theta-wave oscillatory power and theta-peak potential coupling were significantly correlated with nociceptive behavior. Inhibition of vCA1 activity directly induced mechanical nociceptive hypersensitivity, whereas restoration of vCA1 activity relieved chronic pain. This finding suggests that abnormal connections in the hippocampal-limbic system may be involved in the maintenance of the affective component of nociception (Wang et al., 2024). Patients with chronic widespread pain had significantly higher peak resting-state alpha wave frequency (PAF) than patients with chronic back pain, reflecting specific alterations in cortical resting-state functional connectivity under different pain phenotypes. This difference may be related to different patterns of functional integration in pain-related brain regions (McLain et al., 2025).

After spinal cord injury, long upstream propriospinal neurons (LAPNs) have an increased density of axonal collateral branches near the area of injury, along with a dorsal-ventral shift in axonal orientation. This anatomical remodeling may lead to abnormal intersegmental signaling in the spinal cord involved in the maintenance of nociceptive sensitization (Brown et al., 2024). The cerebellum regulates the affective and cognitive components of nociception through extensive connections to the sensorimotor cortex, limbic system, and cognitive regions. Electrophysiologic study has shown that cerebellar injury or abnormal function is closely associated with affective deficits in chronic pain, suggesting a critical integrative role in the multidimensional experience of pain (Li C. N. et al., 2024).

### 4.4 Electrophysiological frequency characterization as an important basis for pain mechanism resolution and frequency-specific modulation

The keyword “frequency” will explode to 7.32 after 2020, and high-frequency cited literature Woolf (2011) confirmed that the frequency of abnormal discharge of DRG neurons is positively correlated with pain behaviors, and Devigili G (2008) found that the frequency of abnormal discharge in patients with small-fiber lesions can be used as a diagnostic biomarker. The EP technique based on multidimensional frequency characterization can not only accurately resolve the pain neural coding mechanism, but also realize the precise identification and dynamic monitoring of pain level. Based on this, the development of frequency-specific neuromodulation technology significantly improves analgesic efficacy and promotes the transition of pain diagnosis and treatment mode to a dynamic and individualized direction.

It has been found that the nonlinear features of electroencephalogram (EEG) in the theta/alpha/beta frequency bands are effective in differentiating the severity of chronic NP (Zolezzi et al., 2023). In a neuropathic pain model, the synchronized activity of DRG neurons appeared within hours after injury, earlier than the enhancement of cortical low-frequency oscillations, revealing a mechanism of cortical synaptic remodeling mediated by purinergic signaling (Chen et al., 2023). After spinal cord injury, patients showed significant enhancement of frontal-occipital and temporal–parietal functional connectivity in the beta/gamma frequency bands, and pain further exacerbated the connectivity within the frontal lobes. Brain network analysis based on phase-locked values (PLV) showed significant clustering differences in the theta/alpha/beta frequency bands (Xu et al., 2023).

In terms of precision intervention, 10 Hz rTMS stimulation of the motor cortex immediately reduces the laser-evoked P2 amplitude and enhances the alpha oscillatory power in sensorimotor areas, whereas dorsolateral prefrontal lobe (DLPFC) stimulation produces delayed analgesic effects by prolonging the default mode network-associated microstate C duration (Li X. et al., 2024). High-resolution SCS (HR-SCS) significantly enhanced evoked electromyographic (EMG) responses in lower limb muscles at the T6/T9 level through a vertical tripolar configuration with spatial selectivity superior to that of conventional electrodes (Berwal et al., 2024).

## 5 Conclusion

In this study, we have mapped the whole chain of knowledge in this field from basic research to clinical application through bibliometric visualization for the first time. The central role of the “frequency” feature in the analysis of pain mechanisms and precise intervention has been discovered, which provides a theoretical basis for the development of diagnostic and therapeutic devices based on electrophysiological signal features. We have revealed the emerging research direction of “dynamic pain connectome,” which provides an entry point for multidisciplinary cross-fertilization. Future research should focus on technology standardization and multimodal data fusion: to unify electrophysiological parameters such as stimulation frequency and recording sites; to accelerate the clinical translation of cutting-edge technologies such as single-cell recording and optogenetic modulation. In addition, it is also necessary to strengthen the “connectivity”-oriented pain loop research and develop personalized analgesic solutions based on connectomics, which will help to achieve precise pain diagnosis and treatment based on electrophysiological phenotypes.
